# Missing something? A scoping review of venous thromboembolic events and their associations with bariatric surgery. Refining the evidence base

**DOI:** 10.1016/j.amsu.2020.08.014

**Published:** 2020-08-17

**Authors:** Walid El Ansari, Kareem El-Ansari

**Affiliations:** a Department of Surgery, Hamad General Hospital, 3050, Doha, Qatar; b College of Medicine, Qatar University, Doha, Qatar; c Schools of Health and Education, University of Skovde, Skövde, Sweden; d Volunteer, Hamad General Hospital, Hamad Medical Corporation, 3050, Doha, Qatar

**Keywords:** Post-operative, Venous thromboembolism, Pulmonary embolism, Porto/ mesenteric venous thrombosis, Upper extremity, Bariatric surgery, Policy

## Abstract

**Background:**

Venous thromboembolic events (VTE) post-bariatric surgery (BS) lead to morbidity and mortality.

**Methods:**

This scoping review assessed whether reported VTE post-BS could be under/over-estimated; suggested a possible number of VTE post-BS; appraised whether VTE are likely to decrease/increase; examined BS as risk/protective factor for VTE; and mapped the gaps, proposing potential solutions.

**Results:**

VTE appears under-estimated due to: identification/coding of BS and VTE; reporting of exposure (BS); and reporting of outcomes (VTE). The review proposes a hypothetical calculation of VTE post-BS. VTE are unlikely to decrease soon. BS represents risk and protection for VTE. Better appreciation of VTE-BS relationships requires longer-term strategies.

**Conclusion:**

VTE are underestimated. Actions are required for understanding the VTE-BS relationships to in order to crease VTE by better-informed prevention strategy/ies.

## Highlights

• Venous thromboembolic events (VTE) post-bariatric surgery (BS) lead to morbidity and mortality.
• VTE post-BS appears under-estimated due to identification, coding and reporting of both the exposure (BS) and outcomes (VTE).
• The review proposes a hypothetical calculation of VTE post-BS. VTE are unlikely to decrease soon.
• BS represents risk and protection for VTE.
• Better appreciation of VTE-BS relationships requires longer-term strategies.

## 1 Introduction

Morbid obesity is associated with various co-morbidities, including chronic venous insufficiency [1]. Bariatric surgery (BS) is an effective strategy with a good safety profile to achieve meaningful and sustainable weight loss [2]. However, BS carries potential risks and possible complications, of which venous thromboembolic events (VTE) are a significant cause of morbidity and mortality [1,3]. The term VTE includes deep vein thrombosis (DVT) and its life-threatening complication, pulmonary embolism (PE); it is a substantial cause of sickness and death among hospitalized and postoperative (post-op) patients [4,5]; and is a primary reason for early readmission (5.31% of readmissions <30 days after BS) [6].

Obese patients are in a hypercoagulable state, and BS puts them at high VTE and PE risk [7]. Many reasons contribute to this. Morbid obesity diminishes mobility, leading to conditions associated with VTE (hypertension, diabetes, venous stasis, obstructive sleep apnea) [[8], [9], [10]] and there often is venous stasis and a chronic inflammatory state [11]. Surgery in obesity is also associated with longer operative time which in turn independently increases VTE risk; and the complexity of some BS (creating 2 separate anastomoses) adds opportunity for endothelial vessel damage and VTE for obese patients already at risk of thrombosis [[12], [13], [14], [15]]. Likewise, concurrent conditions (e.g. hernia repair) and complications (e.g. anastomotic leaks) [16] contribute to prolonged immobility that increases the risk of VTE. In addition, major bleeding (more likely in gastric bypass vs sleeve gastrectomy) that necessitates blood transfusion might also significantly increase the risk of VTE (Odds Ratio 4.7; 95% CI 2.9–7.9) [17]. Conversion to open surgery had the greatest impact on VTE, suggesting that perioperative factors like exposure to tissue factor by larger wounds have great bearing on VTE [18].

Notwithstanding, VTE represent a preventable cause of mortality [19]. Given the obesity epidemic and the frequency of BS as a management strategy, in order to prevent morbidity and mortality, the VTE-BS relationship requires clearer understanding. These considerations inspired the current review.

## 2 Methods

The aim of a scoping review as undertaken in this manuscript is not to find, retrieve and include every single paper published on the topic. Rather, the aim is intended to be broader, focusing on exploratory reconnaissance searches of the relevant literature to determine key characteristics involved in the subject at hand, map potential gaps, and illustrate pertinent examples. Such goals agree with the scale of what a scoping review can achieve, which include identifying types of existing evidence in a given field, key characteristics related to a certain topic, or knowledge gaps [20]. Scoping reviews are useful for answering broad questions, e.g., “What information has been presented on this topic in the literature?” and for collecting and appraising information before undertaking a systematic review [20]. A scoping review is specifically advantageous when the information on a topic has not been comprehensively reviewed or is complex and diverse [21], as the broad scope of the information makes using formal meta-analytic methods difficult, if not impossible [22].

VTE after BS is a complex and diverse topic requiring broad coverage. Hence the scoping review was selected to examine this relationship. We employed a six-step rigorous framework used for scoping reviews (highlighted below) and applied its criteria that comprised: identifying the research question/s; identifying relevant studies; study selection; charting the data (the data extraction process); collating, summarizing, and reporting the results; and (optional step 6), a consultation exercise [23].

### 2.1 Research questions

In order to assess VTE after BS, the current review ‘scoped’ the published literature to answer six related questions related to a public health problem: 1) How frequent are VTE post-BS?; 2) How ‘accurate’ are such reported frequency/ies?; 3) What is a possible/plausible (although hypothetical) number of VTE post-BS and its clinical impact?; 4) Is the frequency of VTE post-BS likely to decrease in future?; 5) Is BS a risk factor or a protective factor for VTE?; and, given the answers to the first 5 questions, 6) What is a possible way forward?

### 2.2 Identifying relevant studies

#### 2.2.1 Information sources

The review team searched electronic databases including PubMed, MEDLINE, Embase, CINAHL, Web of Science, Scopus, as well as Google scholar for published articles of all types of thromboembolic events and their associations with any type of bariatric surgery relevant to answering these research questions.

#### 2.2.2 Keywords and search terms

The keywords used were “bariatric surgery” [in Title/Abstract]. The medical subject headings (MeSH) terms used were bariatric surgery [All Fields] AND (“thromboembolic" [MeSH Terms]; bariatric surgery [All Fields] AND (“thrombosis" [MeSH Terms]; bariatric surgery [All Fields] AND (“embolism" [MeSH Terms]; bariatric surgery [All Fields] AND (“thrombosis AND embolism" [MeSH Terms]; bariatric surgery [All Fields] AND (“postoperative” AND thrombosis” [MeSH Terms]; bariatric surgery [All Fields] AND (“postoperative” AND embolism” [MeSH Terms]. As more features related to VTE and to BS were uncovered from the retrieved literature, additional searches were devised and undertaken in order to capture and retrieve literature pertinent to the uncovered features.

### 2.3 Study selection

#### 2.3.1 Inclusion criteria

Study design: original studies; Language: published articles in English language; Time period: original studies published from January 01, 1990 through March 30, 2020; Interventions: published articles that assessed “bariatric surgery”; and, Participants: published articles enrolling patients of any age, gender, and ethnicity.

#### 2.3.2 Exclusion criteria

Studies that did not include bariatric surgery, thrombosis or embolism; and, studies in patients with inherited coagulation abnormalities presenting as thrombotic risk (e.g. FV-Leiden) or bleeding tendency (hemophilia).

### 2.4 Charting the data

Data items extracted: we extracted items that would be relevant to answering the research questions: reported incidences of all types of VTE post-BS; the likelihood of whether such incidence could represent under/over-estimation of the problem; data that would assist to hypothesize a possible estimate of VTE post-BS based on available published data; evidence that could aid in forecasting whether VTE will likely decrease or increase in future; and information on whether BS a risk/protective factor for VTE.

### 2.5 Collating, summarizing, and reporting the results

We collated and summarized the results and report it below. Based on the emergent findings, the review mapped potential gaps that, if addressed, could present opportunities for probably advancing the field and understanding of VTE after BS, and proposed potential solutions as a way forward.

### 2.6 Consultation exercise

The review and its findings were presented to two experts (senior consultants), one in the bariatric field and the other in the intensive care field (with particular expertise in VTE) in order to provide their insights to inform and validate the findings from the current scoping review.

## 3 Results

### 3.1 How frequent are VTE post-BS? Reported incidence

For this first question, we retrieved reported incidences of VTE post-BS from the literature. Across 304,515 BS patients, in-hospital rate of VTE was 0.17%, and open gastric bypass had the highest rate (0.45%) [3]. DVT can occur in >20% of BS patients [24,25], and there is a 0–3.4% incidence of PE after BS [26,27]. In Brazil, 3 out of 53 BS patients developed post-op distal venous thrombosis (7.5%), but none had clinically manifested PE [1]. Others found an incidence of symptomatic DVT and PE post-BS of 0%–5.4% and 0%–6.4% respectively [[28], [29], [30], [31]].

Such variations in reported incidences of VTE and PE could be due to many challenges (e.g., whether all VTE and PE are symptomatic/clinically manifested, if and how a screening for VTE took place, time frame in which VTE appear/are diagnosed, relationships between laparoscopic BS and VTE, others). For instance, only 1 in 3 VTE in BS becomes clinically significant [32]; 80% of VTE occurred post-discharge (most VTE appeared within the first month after surgery) [33,34]; and it is not clear whether laparoscopic BS has reduced risk of VTE [35]. Understanding the probable reasons for the disparities of reported incidences of VTE and PE post-BS is not uncomplicated, and has to do with the levels of ‘certainty’ of the reported frequency/ies. This prompts the second question of the current review.

### 3.2 How ‘accurate’ is the reported frequency/ies of VTE events post-BS? Uncertainties

For question two, we assessed the literature for potential causes that could result in under/over reporting of VTE post-BS. Taking the 2016 International Federation for the Surgery of obesity and metabolic disorders (IFSO) Survey as an example, the total BS procedures undertaken in 2016 alone was 685,874 [36]. Assuming a conservative 0.17% (in-hospital) rate of VTE [3], applied to 685,874 BS [36], then a cautious hypothetical estimate of VTE would be generated for 2016. Such cautious ballpark figure (computed on only in-hospital VTE) [3] is likely to be a considerable underestimate due 3 groups of challenges detailed below.

#### 3.2.1 Identification: Coding of exposure (BS procedures) and outcome (VTE events)

The number of BS may be underreported due to an imperfect sensitivity of coding for procedures [37]. For PE, review and re-abstraction of hospitalizations indicated that 92% of codable cases for PE were on the abstract [38]. Discharge codes for DVT showed that 92% of coded cases of idiopathic DVT were valid [39]. Limitations of administrative databases include accuracy in coding and data input [40]; and as BS data is linked to diagnoses in primary and secondary care, accuracy of outcomes and diagnoses depends on data entry and transcription [41]. Likewise, using administrative diagnoses coded in claims data to categorize patients as overweight/obese may underestimate the prevalence of those conditions [42].

Coding and reporting mistakes may occur in the BS Data Files [43]; and the 2015 Metabolic and Bariatric Surgery Accreditation and Quality Improvement Program (MBSAQIP) database has completeness, accuracy, and consistency issues, and needs standardized coding for complications [44]. Quantifying the validity of diagnosis codes for obesity and other prognostic factors is critical to interpret surgical outcomes [42]. This suggests a slight underestimation of reported VTE post-BS.

### 3.3 Reporting of exposure (BS procedures)

#### 3.3.1 Completeness, registry vs estimated data, representativeness, non-IFSO members

The 2016 IFSO survey has “data from 58/62 (94%) IFSO societies (except Panama, Paraguay, Philippines, Serbia), and 27 out of 58 (45.8%) reported information from their own national registries while the remaining national societies provided estimated data, although they declared a completeness of data of about 80%" [36]. Similarly, the MBSAQIP captures all surgeries performed at accredited centers, but not all BS done [43]. In addition, as non-IFSO members seem not included in the IFSO survey [36], it is uncertain whether additional numbers of BS globally were not represented in the IFSO estimate. Such factors are likely to slightly underestimate the frequency of VTE.

#### 3.3.2 Private sector

Significant number of BS patients pay cash [43]. “Endoscopic and surgical procedures performed in private not academic institutions are usually not reported; therefore, the reported number is probably underestimated” [36]. With no provision of an extent of such underestimation, the examples below might help.

*Reimbursement*: in New Zealand, the annual BS volume increased in the public sector with similar rise in private sector, and private BS amount to 70.2% of total BS [45]. In Australia, BS in public sectors are publicly funded, but most BS occur in the private sector, and Medicare only reimburses surgical costs in the private sector [46]. In Japan, bypass surgeries are not covered by public insurance, but are done in some private hospitals [46].

*Some lack of mandatory reporting*: in the USA, as ambulatory surgery centers can offer lower prices for sleeve gastrectomy (SG) and do not have to report discharge data to the state, databases could miss significant percentages of BS [43].

*Waiting times*: could amount to years in BS [47]. In Singapore, surgery waiting time in public hospitals is longer than in private hospitals; patients at private hospitals receive no Ministry of Health subsidies, but have swift appointments/short waiting time for BS [46].

*Post-op follow up criteria*: in Australia, private and public sectors have different criteria for post-op follow up [46]. The Bariatric Surgery Registry (BSR) in Australia monitors outcomes of public and private BS, but about 50% of surgeons registering a BS interest with the BSR actually contribute to the registry [46].

Hence globally, substantial BS happens in the private sector (for domestic residents due to long waiting lists, cost reimbursement protocols, procedure available only in private sector, etc.). It is not evident whether/how such private sector BS volume and complications are captured, suggesting that this could result in quite an underestimation of reported VTE post-BS.

#### 3.3.3 Bariatric tourism

This is travel for the primary purpose of receiving medical treatment. At least 2% of the global BS is provided for medical tourists [48]. Most data is from academic medical centers [49], hence private practices, where most bariatric tourism happens, would not be included in these figures. However, some data might aid in speculating the magnitude of this aspect of BS.

The complication rate for bariatric medical tourism is substantially higher than the local rate (42.2–56.1% vs 12.3%), suggesting that locally conducted surgery has lower complication rate than that of bariatric medical tourists [50]. An international survey (93 surgeons) showed that 64 operated on foreign patients (total 3740 operations), where one surgeon provided BS strictly for tourists, and 3 surgeons treated foreign patients in >50% of their BS performed [48]. Only 80% of these surgeons recommended bariatric check-ups in the country of origin [48], despite that follow-up provides better outcomes [51]. About 20% of the surgeons reported that these tourists experienced complications (VTE were the most common), which was suggested to be due to air travel; and there was no data as to whether the episodes of VTE were related to PE [48]. It is also not clear whether some bariatric surgeons could be over-conservative with the use of anticoagulation for bariatric tourists due to the fear of bleeding as a complication for a tourist who would be travelling, and hence not under the surgeon's direct supervision. Most bariatric tourists do not receive coordinated, long-term post-op care from foreign health care services [52]. In Canada, there was no formal tracking system to identify Canadian bariatric tourists; and no hospital systems to distinguish complicated medical tourists from local patients, other than clinic records or chart review [50]. With globalization of health care, increasing number of patients continue to travel for medical care [53]. It is remains to be understood how such volumes of global BS tourism and their complications (e.g. VTE) are documented. This suggests an underrating of BS and of VTE.

### 3.4 Reporting of outcomes (VTE)

#### 3.4.1 Affiliation of surgeon

“The Bariatric Surgeon's Qualifications” consensus highlights that bariatric surgeons must be properly qualified general surgeons, had preceptorship with a well-experienced, qualified bariatric surgeon, and be an IFSO member; Prior to independently performing primary BS, each surgeon needs to meet minimal standards that include affiliation with or application for membership in IFS0/IFS0 Adhering Body [54,55]. Surgeon experience is a cause for hospital readmission in BS [6]. The inverse correlation between post-op complications and surgeon experience [56] led to the establishment of BS practice guidelines [57], Centers of Excellence, and public availability of surgical outcomes [49]. For BS complications, “the variability between centers is, at best, related to intervening variables which are poorly quantifiable; this makes comparisons by means of a complication standard potentially inaccurate” p. 498 [54]. If affiliation is conceptualized as an ‘intervening variable’, then the relationship between non-affiliation and VTE rate might require understanding, particularly that non-IFSO members are not included in the IFSO survey [36].

#### 3.4.2 Accreditation of institute/center

A medical institution's experience reduces the complications of obese patients via integrated care, adequate preparation, standardized surgical procedures, early detection and management of complications [58]. Mortality and morbidity of patients operated on in Centers of Excellence were much less than non-accredited centers [59]. Accreditation was associated with better BS outcomes, independent of volume status [60]. About 90% of the BS reviewed were done at accredited centers [49], and some databases lacked data on where the BS was done [42], making it unfeasible to ascertain whether a given BS was at an accredited center or otherwise.

Texas performs 10% of BS in the USA, where 13%–20% of SG procedures were performed at non-accredited centers [43]. ‘If this is a trend that is applicable on a national level, we may be missing nearly one in six cases of bariatric surgery” [43]. There were 84 Accredited Centers in Texas (2017), but 171 centers were performing BS [43]. Another database had data from 153 facilities, but this may still underestimate the true number of BS done in Texas [43]. BS at accredited centers could have fewer complications (e.g. VTE) than at non-accredited centers.

#### 3.4.3 Lack of requirement of mandatory reporting

Hospitals exempt from reporting are federal hospitals and those that do not get reimbursed from government sources [43]. In the USA, as ambulatory surgery centers do not have to report discharge data to the state, there could be another significant percentage of bariatric cases (and complications) that current databases are missing [43].

#### 3.4.4 Possible biased reporting (reputation)

This is a hypothetical proposition that, in some countries, a likelihood that the documentation of VTE particularly, or complications generally, might be subject to some biased reporting due to any probable ‘reputation-harming’ reason/s, particularly in the private sector. Just as acute myocardial infarction and pneumonia are publicly reported outcomes that impact a hospital's reputation and funding [61,62], a BS program's reputation might similarly be impacted by its readmission rate [6]. In some parts of the world, avoiding 'dents' to reputation might impact on practice trends. Given the revenues of obesity management [48], along with the reality of present-day healthcare economics, it might be fairly ‘down-to-earth’ to consider that this might occur in very few parts of the world. This suggests a slight underreporting of VTE post-BS.

#### 3.4.5 Symptomatic vs asymptomatic VTE

VTE are identified based on testing aimed at symptomatic patients or clinical suspicion of disease, although some studies performed imaging to detect asymptomatic DVT [18,63], and it is unclear whether asymptomatic DVT is included in VTE statistics. Whilst VTE as represented by symptomatic VT/PE is likely to be accurate because this does not require subjective evaluation [40], only 1 in 3 VTE in BS becomes clinically significant, leaving the true incidence of post-op VTE grossly underestimated [32]. Indeed, PE was found in 8 of 10 deceased patients after BS; only in 2 cases there was clinical suspicion for PE [64]. More than 50% of post-BS patients who develop VTE had no clinical symptoms of VTE or radiologic evidence at discharge [18]. However, screening all patients is impeded as colour Doppler ultrasound has low sensitivity for detecting asymptomatic DVT in obese patients [65,66].

Such symptomatology is also related to the point below (in-hospital vs after-discharge VTE) and proposed the question as to whether VTE “occurring after discharge actually do not form until the patient is discharged, or are they just not diagnosed until after discharge?” p. 1141 [67]. Reporting only symptomatic VTE post-BS probably contributes a sizeable underestimation of the frequency of this event.

#### 3.4.6 Variations of time periods (in-hospital vs after-discharge VTE)

The duration (and magnitude) of post-surgery risk for VTE remains unclear in some instances as to whether a reported rate represents in-hospital vs longer-term VTE. VTE incidence during the index surgical hospitalization was 0.88%, a cumulative rate increasing to 2.17% at 1 month and 2.99% by 6 months post-surgery [68]. Others found a VTE cumulative incidence at 7, 30, 90, and 180 days of 0.3, 1.9, 2.1, and 2.1%, respectively (180-day 95% CI 0.7–3.6%) [5]. Most VTE occur within 30 days post-BS [34]; mean time to diagnosis of VTE post-BS was 24 days [69]; and 74%–80% of VTE appeared after discharge [68,70].

Over 50% of post-BS patients who develop VTE had no clinical symptoms of VTE or radiologic evidence at discharge [18]. VTE after discharge are serious, particularly after BS: and although the prevalence of post-discharge VTE was 0.29% among BS patients and the entire cohort's mortality was 0.1% [71,72], those experiencing post-discharge VTE had higher mortality (2.60%) [33]. Standardization of the time periods of data reporting in this domain would be beneficial, as given the trend of shorter hospital stays for surgery patients, reporting only in-hospital rates will inevitably underestimate VTE post-BS.

#### 3.4.7 Other thromboembolic events not often explicitly included in VTE statistics

##### 3.4.7.1 Upper extremity DVT

Data on upper extremity deep vein thrombosis (UEDVT) and its management are limited [11]. UEDVT represents <10% of VTE, but is associated with higher incidence (3–12%) of asymptomatic PE [11]. Objective findings of PE were found in 36% of UEDVT patients [73,74]. Others reported post-BS UEDVT in 5 of 1503 patients; but only 1 case was associated with the use of an indwelling catheter, and another had a history of VTE [11].

##### 3.4.7.2 Porto/mesenteric venous thrombosis

Porto/mesenteric venous thrombosis (PMVT) complicates about 1% of SG [67], and has also been reported after laparoscopic Roux-en-Y gastric bypass and laparoscopic adjustable gastric banding [75,76]. In 2014, SG was the most performed BS, comprising >50% of all primary BS in 2016, and had the highest increase rates in recent years [36,77,78]. Post-SG PMVT incidence is 0.37%–1% and is increasing with SG's popularity [79]; and a 0.1%–1.81% PMVT incidence has been observed post-BS including laparoscopic BS [[80], [81], [82]]. Research has reported a 1.61% PMVT mortality rate [82]. It is unclear if this increased incidence is due to the increasing frequency of LSG, or if LSG carries independent PVT risk [83]. In addition to the hypercoagulable state of obesity, the mechanical/thermal effects of laparoscopic surgeries could be associated with PMVT [84,85]. Nevertheless, PMVT's true incidence is not known, is underestimated [86], and there is infrequent reporting of PMVT as a BS complication [87], despite its life-threatening consequences. PMVT appears not routinely included when reporting VTE post-BS; and such non-inclusion will underestimate the VTE after BS. This introduces the third question: “What is a possible plausible number of VTE post-BS, and its clinical impact?"

### 3.5 Estimation of a possible number of VTE post-BS

Estimating a ‘true’ VTE frequency post-BS is not easy. Across antireflux surgery, gastric bypass, appendectomy, and cholecystectomy, ‘the true incidence of VTE is unknown’ [40]. When the above factors are forecasted to consider the upward yearly trends of BS globally, it might be fair to speculate that a ‘true’ rate of VTE is likely to be more than the estimated patients per year globally (suggested above, section 3.2). Indeed, post-BS, the rate of VTE ranges between 0.2% and 3.8% [40,68,[88], [89], [90]], DVT ranges between 0% and 5.4% [28,29], and PE ranges between 0% and 6.4% [30,31]. Using a middle value (3.8%) of these reported rates, and then decreasing this 3.8% by another say, 2% to become 1.8% (as a guard against possible over inflation of VTE estimate), then applying it on 685,874 BS in 2016 [36], a potential hypothetical number of VTE events (morbidity) post-BS in 2016 could be generated. If one further hypothetically considers that 1 in 3 VTE in BS becomes clinically significant [32], then an additional considerable number of individuals could suffer non-clinically significant VTE post-BS, representing the part of the iceberg beneath the waterline, a potentially dangerous proportion of patients that could, due to un/known further change, progress to clinically significant VTE. If one then systematically includes ‘educated guesses' of hypothetic additional effects of other causes of underreporting of exposure (BS) and outcome (VTE) (enumerated above), the numbers will again increase, suggesting a significant underestimation of current rates of VTE. Although this hypothetical frequency of VTE represents many patients, the next step of assessing readmissions, mortality, clinical significance and impact due to post-BS VTE is not straight forward.

### 3.6 Clinical significance and impact of VTE events

VTE represent considerable clinical significance. In primary BS, VTE were the third complication (0.3%, more common than leak 0.2%) [91] and had the greatest effect on readmission and mortality rates, hence reducing post-BS VTE has potential to lower these rates [91]. PE is a leading cause of death post-BS, and a common autopsy finding [64,[92], [93], [94]]. Among BS patients, 0.4% developed VTE, 0.2% had PE, and 0.02% died of VTE [95]; and others reported 0.33% VTE rate, including 51 patients with PE, and there were 8 associated deaths (8.6% case fatality rate) [16]. The 30-day post-discharge VTE incidence was 0.29%, and among those having post-discharge VTE, mortality increased 28-fold [33]. Likewise, the incidence of fatal PE is uncertain. PE was found in 8 of 10 deceased patients post-BS; in 2 cases only, there was clinical suspicion of PE [64]. PE after BS has high mortality [92], particularly post-discharge mortality [93,96]. Autopsy found PE as leading cause of death in 20.7% of deceased patients after BS [97]; and PMVT has 1.61% mortality [82].

If one takes a middle-boundary of 3.8% post-BS VTE rate [40,68,[88], [89], [90]] and then decreases it by another say 2% to become 1.8% (as a safeguard against possible over inflation of VTE estimate), and apply it to 685,874 BS in 2016 [36], and then take the 8.6% case fatality rate [16], reduce it by half to 4.2% (as a safeguard against over-inflation) and apply it, then a hypothetical number of deaths would result for 2016. This hypothetical number does not consider additional non-clinically significant VTE or other causes of underreporting of exposure (BS) or outcome (VTE).

Thus far, the literature suggests that VTE appears to be more common than meets the eye, with important clinical impacts. This raises question four: “Is the frequency of VTE post-BS likely to decrease in future?” Some features below might help to answer this question.

### 3.7 Are VTE post-BS likely to decrease in future?

#### 3.7.1 Increasing numbers of BS procedures are forecasted

As obesity increases, demand for BS will increase [98], as BS is now safer with less complications [99]. SG increases significantly yearly; and BS shows an upward trend with time. In Brazil, BS increased exponentially, due to high demand of the obesity epidemic affecting Brazil and the world [100,101]. In Australia, BS increased fivefold (2003–2008) [47]. In Ontario, Canada, BS showed 858% change (2006–2007 vs 2012–2013) p. 25 [102]. England, France and Sweden also experienced increases in BS recently [[103], [104], [105]]. Notwithstanding, BS is decreasing in some countries [106]. Nonsurgical weight-loss (diet, exercise, psychological support, pharmaceuticals) has limited/un-sustained benefit for most people [49], and minimally invasive techniques, media coverage, and patient satisfaction lead obese individuals to BS as a reliable option [107].

#### 3.7.2 Insufficient firm evidence of superiority of laparoscopic approach

The risk of VTE among BS is persistent even with laparoscopy [69], and VTE are now frequent with widespread laparoscopic BS [108], despite that laparoscopic BS was more likely to be at urban higher-volume hospitals [109]. ‘The true incidence of VTE after laparoscopic compared with open surgery is unknown’ [40]. The risk of reoperation is not different between laparoscopic and open BS [110,111]. During laparoscopic BS, increased intra-abdominal pressure decreases venous flow in the legs, increases venous stasis [1,112,113], and pneumoperitoneum has negative effect on hemodynamics during laparoscopic compared to open BS [114]. While the effects of laparoscopic and open gastric bypass were essentially the same [115], with similar risk of reoperation [116], others found increased reoperation in open vs laparoscopic BS but significantly lower mortality [109], hospital stay [116], and incidence of VTE [117] for laparoscopic BS. This is not unique to BS; in rectal cancer surgery, no firm conclusions exist on the superiority of laparoscopic vs open approach [118,119].

#### 3.7.3 Increasing numbers of conversions to open surgery

Conversion from laparoscopic to open surgery exhibits wide discrepancies, ranging from 0.8% to 4.1% [18,[120], [121], [122], [123], [124], [125], [126]]. The relative risk of VTE after laparoscopic vs open surgery is unknown [40]. For appendectomy, antireflux surgery, cholecystectomy, and gastric bypass, compared with laparoscopic, open procedures had significant risk for VTE [40]. Conversion to open surgery had the greatest impact on VTE, with a very high relative risk (RR = 20.2) [18], and patients who require conversion of primary laparoscopic RYGBP should know the substantially higher VTE risk and receive aggressive peri-op thromboprophylaxis [18].

#### 3.7.4 Insufficient firm evidence that VTE after BS is decreasing

The risk of VTE among BS patients is persistent despite aggressive prophylactic anticoagulation [69]. Others noted that the overall rates of VTE after BS increased 2.5-fold (0.08%–0.215%, 2008 vs 2012) [99]. The risk of acute care use for VTE was transiently increased after BS in the immediate post-op period, and was back to baseline level within a few months but not further reduced during a 2-year follow-up [127]. Given a projected increase in BS, this suggests that probably escalating numbers of VTE post-BS might be encountered every successive year.

##### 3.7.4.1 Prophylaxis of VTE and its effectiveness

There appears no consensus on the standard of care for prophylactic agents, dosing, timing, or duration in BS [128]. Post-BS prophylaxis against VTE has been largely adopted using data from general surgery, with limited optimization of preventive considerations specific to BS [129]. Decisive data regarding the most effective and safe prophylactic methods for VTE in BS are lacking [25,130], and incidence of major bleeding seems to increase using weight-adjusted doses of heparin, with no advantage in terms of VTE reduction [117].

##### 3.7.4.2 Stratification of risk

Prediction tools remain limited in establishing a patient's risk pro-file for VTE, rendering prophylaxis guidelines difficult to implement [16,131]. About 33% of patients with post-BS VTE did not have additional risk factors (apart from the risk factors of obesity and abdominal surgery) [18]. This might argue against limiting the use of additional preventive measures against VTE in ‘high-risk’ patients only, as some patients will still remain at risk and thus sub-optimally protected [18].

##### 3.7.4.3 Dosing, timing, and duration

The optimal prevention strategy against VTE is uncertain [4], with no firm evidence for the period through which thromboembolic prophylaxis should be continued post-BS [15], despite that the duration of chemical thromboprophylaxis may be more important than dosing [18]. Although prophylaxis was used in all patients, the incidence of post-BS distal VTE was considerably high [1], with not enough data to recommend the most effective and safe prophylaxis [132]. Interestingly, BS patients on pre-op anticoagulation medications had higher risk of all post-op adverse outcomes, where some research reported that VTE were significantly higher among pre-op anticoagulated patients vs no pre-op anticoagulation (0.68% vs 0.25%, p < 0.001) [133]. Such evidence does not represent firm grounds to speculate that current knowledge of prophylaxis will likely decrease VTE after BS than they currently are.

The literature does not seem to firmly forecast a likely decrease of VTE post-BS in the near future. This stimulates the fifth question: Is BS a risk or a protective factor for VTE? BS exhibits a unique feature in its relationships with VTE.

### 3.8 Is BS a risk factor or a protective factor for VTE events?

#### 3.8.1 Risk factor

The contribution of obesity to the thromboembolic risks of surgery suggests that BS patients have high risk of post-op PE/DVT [134]. BS has high risk of VTE, similar to total hip or knee arthroplasty [5]. For BS patients, the risk profile of VTE is complex with several factors which themselves increase the risk of VTE [135]. The risk of VTE in BS is persistent despite use of laparoscopy and aggressive prophylactic anticoagulation [69]; and thromboembolic complications continue as a main reason for perioperative mortality post-BS [92,97,132].

#### 3.8.2 Protective factor

Very few studies assessed the risk of VTE beyond the first year post-BS [41]. Despite the short-term increase in thromboembolic risk (increased risk for up to 6–12, months after surgery) [68,69,96] the long-term effect of BS on thromboembolic events (overall reduction in risk of VTE associated with weight loss for up to 3 years post-BS) remains greatly unassessed [41,136]. The balance between the known short-term increase in thromboembolic risk due to BS, and the less explored long-term benefit due to weight loss suggests that overall, BS effectively nearly halves the DVT risk in the long term with corresponding decrease in VTE [41]. Across 10.7 years follow-up, the long-term benefit of BS on VTE risk outweighed the short-term risk [41]. There seems to be an ‘offset'.

Hence, the answer to this question seems to be yes to both: BS is a risk and also a protective factor for VTE, subject to the time frame that is examined. Remarkably, the BS-VTE relationship exhibits two opposite (conducive and protective) processes simultaneously: a shorter-term increase in risk of VTE due to BS and obesity, and a longer-term decrease in risk of VTE due to benefits of BS-related weight loss.

This review has limitations. First, the current review attempted to propose several potential estimates (e.g., frequency of VTE after BS, potential numbers of clinically asymptomatic VTE after BS, potential numbers of deaths after VTE), although scoping reviews are undertaken to provide an overview of the available research evidence without producing a summary answer to a discrete research question [23]. Hence, such proposed estimates should be considered with extreme caution as these values are premised on several assumptions, and hence should be interpreted as being purely hypothetical estimates using literature-informed but completely arbitrary cut-offs. A more precise methodology of identification of such values, where available, would be by prospective studies with hard end-points and sufficient follow up. For this reason, a systematic review and meta-analysis of VTE after BS would be advisable. The same caution applies to other values that the current review proposed.

Second, several of the uncertainties uncovered by the review (e.g., identification: coding of exposure and outcome, reporting of exposure and of outcome, etc.) are not intrinsic, inherent or unique to VTE or to BS *per se*, but rather, are frequent sources of underreporting of surgical outcomes generally. Recent reports addressing this point, e.g., the recent consensual international recommendations on surgical outcomes reporting would be useful here as it provides a consensual checklist in an attempt to customize the methodology of outcome reporting in surgery and thus holds potential to improve the reproducibility and comparability of data and to improve quality of care [137].

Third, comparisons of rates of VTE between historic open surgical vs the more contemporary laparoscopic cohorts could be somewhat biased. Modern perioperative surgical care includes multimodal approaches to prevent and/or reduce VTE, e.g. pre-habilitation, preoperative low molecular weight heparins, intermittent mechanical compression of the calves, early mobilization and prolonged VTE prophylaxis after discharge. Such strategies were not always available or prescribed in the era of open BS.

Fourth, in terms of prophylaxis against VTE and its effectiveness, while some research suggests that there appears to be not enough data to recommend the most effective and safe prophylaxis [128], recent work has provided updates for clinical practice guidelines for thromboembolic prophylaxis in bariatric patients [138], as well as other useful information of perioperative complications in morbid obesity [58,139].

Fifth, narrative reviews could be vulnerable to the subjective biases of the review team; hence we were aware and cognizant to outline a balanced view of the findings, and conscious to project objective opinions as suggested by the state of the literature, ‘uncontaminated’ by preconceptions or preconceived notions. The review was not initiated in order to confirm or refute a specific point; rather, it asked questions and attempted to answer them in a neutral manner to the best of the ability of the review team and the available literature with the aim of enhancing the evidence base. Despite these limitations, the current review could be the first to explore, in depth and breadth, a multitude of aspects and variables of an exceptionally complex, diverse and important topic that appears to not have been comprehensively reviewed.

## 4 Now what? Possible way forward

Collectively, the above findings highlight considerable underreporting of incidence of VTE, and hence significance and impact. Better understanding of the problem, risk, and prophylaxis is required in order to determine more accurate rates, establish duration cut offs, guide prophylactic considerations, assist better-informed clinical decision-making, and agree follow-up durations. Such actions and understanding will need longer-term multi-pronged strategies (Table 1):

1. *Training and checks*: to improve the identification aspects of VTE, including coders and data inputs; and to develop guards against missing data, out-of-range values, unrealistic changes in parameters over time, clearly erroneous information, and miscoded information.
2. *Policy and gentle lobbying*: a group of strategies will have to do with policy e.g. lobbying for modification of policy to advocate mandatory reporting of discharge data where it is not; or assessments of whether facility accreditation might be requirement for coverage of BS.
3. *Motivation, encouragement, incentives, support and creativity*: e.g. to encourage national societies to provide actual (not estimated) data as complete as possible; generate creative means/incentives to attract non-IFSO members to contribute their data; encourage unaffiliated/independent BS surgeons to report statistics via organized channels; support surgeons registered in e.g. Bariatric Surgery Registry to contribute to the registry (Australia); motivate or mandate medical tourism providers to report exposure and a range of outcomes; motivate bariatric surgeons to affiliate with international or national BS associations; and support nonaffiliated surgeons to report outcomes without prejudice or loss of autonomy.
4. *New habits (ways of doing business), criteria, codes and consensus*: e.g. to establish uniform sets of criteria for post-op follow up for private and public sectors; develop codes in hospitals' emergency department/inpatient wards to easily distinguish complicated medical tourists from local patients; develop criteria for asymptomatic VTE; report VTE events strictly by time period in which they occurred (if known); ensure high clinical suspicion for PMVT, and regular and consistent reporting of PMVT when it occurs post-BS along with other DVT events that occur post-BS; analysts to be cautious when interpreting patient care events; develop process and outcome indicators; consensus on dosing guidelines for pharmacologic prophylaxis in obese patients who are already at increased risk for VTE [140]; and early identification of post-op problems and timely intervention/s [6].
5. *Development, refinement and validation of tools*: e.g. validate and refine risk calculator/s and scoring systems to detect high risk individuals prone to asymptomatic VTE; validate multiple diagnostic codes required for high quality research on post-BS complications; better stratification of DVT risk in BS patients [27] and at discharge [141,142]; and refined cut-points on estimated risk of post-discharge VTE to guide extended pharmacoprophylaxis [33].
6. *Identification and validation of best practice*: reviews, systematic reviews and metanalyses would provide important information. Future research could focus on optimizing pharmacological and non-pharmacological prevention strategies considering the risk profile of BS. This could include pre-operative metabolic optimization and preparation of patients, as well as pre-rehabilitation (e.g. improved general muscle strength, coughing, walking distance or stairs) that could contribute to lower the risk of VTE.

**Table 1:** Potential reporting of VTE: challenges, effects and potential solutions.

Issue	Effect a	Examples of Potential solutions
Identification	**+**	Enhance knowledge and experience of International Classification of Diseases to coders [143]
Coding of exposure b, outcome c, obesity		Validate multiple diagnostic codes [143]; Use reliability indicators; Develop guards against missing data, out-of-range values, unrealistic changes in parameters over time, erroneous or miscoded information. Analysts to be cautious when interpreting patient care events [145]
Reporting of exposure (BS procedures)		

Completeness, registry vs estimated data, representativeness, non-affiliated members	**+**	Global surveys to motivate/ incentivize societies to submit more complete data, maintain more accurate national registries. Encourage national societies to provide actual (not estimated) complete data. Develop process and outcome indicators; collect comorbidity data occurring within 1 year before index surgical admission [146]. Generate creative means/ incentives to entice/ attract non-affiliated members to contribute their data
Private sector	**+ + +**	Encourage unaffiliated/ independent BS surgeons to report statistics via organized means (without limitations of independence, forced network integration, or infiltration of their market share) (USA) [147]. Advocate mandatory reporting of discharge data where it is not (USA) [43]. Establish uniform criteria for post-op follow up for private and public sectors (Australia) [46]. Support surgeons registered Bariatric Registry to actually contribute to registry (Australia) [46]

Bariatric tourism	**+ +**	In e.g. Canada, out-of-country claims reimbursements, out-of-country in-hospital costs, patient reimbursement for medical tourism is available through local Heath Services [50]
		Develop codes in emergency department/ inpatient wards to distinguish complicated medical tourists from local patients [50]. Medical tourism providers/ travel facilitators to give accurate information to patients about risks [148], and encouraged to report exposure b and outcomes c
Reporting of outcomes (VTE)		
Affiliation of surgeon	**+**	Motivate bariatric surgeons to affiliate (attractive 'affiliation package'). Support nonaffiliated surgeons to report outcomes without prejudice

Accreditation of institute/ center	**+**	Assess if facility accreditation might be requirement for coverage of BS. Ensure codes for whether facility is accredited or otherwise

Lack of mandatory reporting	**+**	Assess modification of policy ± consider incentive strategies to encourage reporting of discharge data

Biased reporting (Reputation)	**?**^d^	Not applicable

Symptomatic vs asymptomatic VTE	**+ + +**	Computed tomographic venography and magnetic resonance venography detect asymptomatic pelvic DVT post-BS, although clinical significance requires more certainty [149]. Low sensitivity of Doppler ultrasound to detect asymptomatic DVT in obese patients [65,66]. Validate and refine risk calculator/s to detect high risk individuals prone to asymptomatic VTE (risk-stratified approach to VTE) prophylaxis. Prevention: Develop and implement pharmacologic thromboprophylaxis in morbidly obese patients undergoing BS to prevent silent DVT, e.g. perioperative low-molecular-weight heparin for 2 weeks post-op with graduated compression stockings is safe/effective to prevent silent DVT post laparoscopic BS [150]
Variations of time periods e	**+ +**	Report VTE events strictly by time period in which they occurred (if known). Prevention: extended 10 day treatment after discharge significantly reduces the incidence of VTE compared to in-hospital treatment only [98]
Other thromboembolism events		
Upper extremity DVT	**+**	Routine inclusion of this entity when reporting VTE post-BS
Porto/mesenteric venous thrombosis	**+**	Acknowledging PVT as a post-BS complication is a necessity, especially for emergency clinicians, surgical residents, general and bariatric surgeons [85]. Requires a high clinical suspicion. Vague presenting clinical features so healthcare professionals need to consider PMVT in the differential diagnosis [151]. Needs to be regularly/ consistently reported when it occurs post-BS and included with any other DVT events that occur post-BS

a possible effect on reported VTE estimate if issue is resolved.

b exposure: BS procedures.

c outcomes: VTE events.

d hypothetical proposition, hence its effects are not estimated.

e reporting of in-hospital vs after-discharge VTE; +: remedy will probably result in slight increase of reported VTE; + +: remedy will probably result in moderate increase of reported VTE; + + +: remedy will probably result in considerable increase of reported VTE.

The above are mere examples. Better understanding of the links between BS and VTE and its attending actions are unlikely to be quick. Rather, these would be incremental, necessitating interprofessional partnerships, and equally requiring science, art, and politics. The hope is for better quality patient care achieved through net gains of a systematic comprehension of the VTE challenge, better appreciation of risk, and better knowledge of the aspects of prophylaxis. Achieving these goals would translate into better statistics on the expected VTE load, more accurate and earlier categorization and pre-op counselling of patients, more informed operative clinical decision making, and better post-op discharge classification of VTE risks and their management. The aspiration is that such new insights will assist in the counselling and assessment of patients in order to decrease VTE after BS.

## Ethical approval

This review analysed data from existing published and unpublished studies. These studies are available in the public domain, ethics approval is not required.

## Funding statement

This study did not receive any funding.

## Author contribution

Walid El Ansari: Data searching, collection and collation, data analysis, writing, reviewing. Kareem El-Ansari: Data searching, collection and collation, data analysis, reviewing.

## Research registration

Research Registry UIN: researchregistry5886.

Link: https://www.researchregistry.com/register-now#home/registrationdetails/5f30197bf0c64f001556d98a/

## Guarantor

Prof Dr Walid El Ansari, welansari9@gmail.com.

## Data statement

The authors confirm that the data supporting the findings of this study are available within the article.

## Consent

No patients were involved in the study.

## Provenance and peer review

Not commissioned, externally peer reviewed.

## Declaration of competing interest

All the authors declare that there are no conflicts of interest regarding the publication of this manuscript.

## References

[1] Bellen Bv, Godoy Ide B., Reis A.A. (2013). Venous insufficiency and thromboembolic disease in bariatric surgery patients. Arq. Gastroenterol..

[2] Pories W.J., Swanson M.S., Macdonald K.G. (1995). Who would have thought it? An operation proves to be the most effective therapy for adult-onset diabetes mellitus. Ann. Surg..

[3] Masoomi H., Buchberg B., Reavis K.M. (2011). Factors predictive of venous thromboembolism in bariatric surgery. Am. Surg..

[4] Geerts W.H., Bergquist D., Pineo G.F. (2008). Prevention of venous thromboembolism: ACCP evidence-based clinical practice guidelines. Chest.

[5] Froehling D.A., Daniels P.R., Mauck K.F. (2013). Incidence of venous thromboembolism after bariatric surgery: a population-based cohort study,. Obes. Surg..

[6] Aman M.W., Stem M., Schweitzer M.A. (2016). Early hospital readmission after bariatric surgery. Surg. Endosc..

[7] Carmody B.J., Sugerman H.J., Kellum J.M. (2006). Pulmonary embolism complicating Bariatric surgery: detailed analysis of a single institution's 24-year experience. J. Appl. Comput. Sci..

[8] Stein P.D., Beemath A., Olson R.E. (2005). Obesity as a risk factor in venous thromboembolism. Am. J. Med..

[9] Ambrosetti M., Lucioni A., Ageno W. (2004). Is venous thromboembolism more frequent in patients with obstructive sleep apnea syndrome?. J. Thromb. Haemostasis.

[10] Ageno W., Becattini C., Brighton T. (2008). Cardiovascular risk factors and venous thromboembolism: a meta-analysis. Circulation.

[11] Safdie F.M., Dip F., Ardila-Gatas J. (2015). Incidence and clinical implications of upper extremity deep vein thrombosis after laparoscopic bariatric procedures. Obes. Surg..

[12] Chan M.M., Hamza N., Ammori B.J. (2013). Duration of surgery independently influences risk of venous thromboembolism after laparoscopic bariatric surgery. Surg. Obes. Relat. Dis..

[13] Wolberg A.S., Aleman M.M., Leiderman K. (2012). Procoagulant activity in hemostasis and thrombosis: virchow's triad revisited. Anesth. Analg..

[14] Gambhir S., Inaba C.S., Alizadeh R.F. (2019). Venous thromboembolism risk for the contemporary bariatric surgeon, Surg Endosc.

[15] Moaad F., Zakhar B., Anton K. (2017). Is LMWH sufficient for anticoagulant prophylaxis in bariatric surgery? Prospective study. Obes. Surg..

[16] Finks J.F., English W.J., Carlin A.M. (2012). Predicting risk for venous thromboembolism with bariatric surgery. Ann. Surg..

[17] Nielsen A.W., Helm M.C., Kindel T. (2018). Perioperative bleeding and blood transfusion are major risk factors for venous thromboembolism following bariatric surgery. Surg. Endosc..

[18] Raftopoulos I., Martindale C., Cronin A. (2008). The effect of extended post-discharge chemical thromboprophylaxis on venous thromboembolism rates after bariatric surgery: a prospective comparison trial,. Surg. Endosc..

[19] Kakkos S.K., Caprini J.A., Geroulakos G. (2016). Combined intermittent pneumatic leg compression and pharmacological prophylaxis for prevention of venous thromboembolism. Cochrane Database Syst. Rev..

[20] Munn Z., Peters M., Stern C., Tufanaru C., McArthur A., Aromataris E. (2018). Systematic review or scoping review? Guidance for authors when choosing between a systematic or scoping review approach. BMC Med. Res. Methodol..

[21] Peters M.D., Godfrey C.M., Khalil H., McInerney P., Parker D., Soares C.B. (2015). Guidance for conducting systematic scoping reviews. Int. J. Evid. Base. Healthc..

[22] Sucharew H., Macaluso M. (2019). Progress notes: methods for research evidence synthesis: the scoping review approach,. J. Hosp. Med..

[23] Arksey H., O'Malley L. (2005). Scoping Studies: towards a methodological framework. Int. J. Soc. Res. Methodol..

[24] Ojo P., Asiyanbola B., Valin E. (2008). Post discharge prophylactic anticoagulation in gastric bypass patient— how safe?. Obes. Surg..

[25] Eriksson S., Backman L. (1997). K.G. Ljungström, the incidence of clinical postoperative thrombosis after gastric surgery for obesity during 16 years,. Obes. Surg..

[26] Cottam D.R., Mattar S.G., Schauer P.R. (2003). Laparoscopic era of operations for morbid obesity. Arch. Surg..

[27] Santo M.A., Pajecki D., Riccioppo D. (2013). Early complications in bariatric surgery: incidence, diagnosis and treatment. Arq. Gastroenterol..

[28] Quebbemann B., Akhondzadeh M., Dallal R. (2005). Continuous intravenous heparin infusion prevents peri-operative thromboembolic events in bariatric surgery patients. Obes. Surg..

[29] Scholten D.J., Hoedema R.M., Scholten S.E. (2002). A comparison of two different prophylactic dose regimens of low molecular weight heparin in bariatric surgery,. Obes. Surg..

[30] Escalante-Tattersfield T., Tucker O., Fajnwaks P. (2008). Incidence of deep vein thrombosis in morbidly obese patients undergoing laparoscopic Roux-en-Y gastric bypass. Surg. Obes. Relat. Dis..

[31] Kardys C.M., Stoner M.C., Manwaring M.L. (2008). Safety and efficacy of intravascular ultrasound-guided inferior vena cava filter in super obese bariatric patients. Surg. Obes. Relat. Dis..

[32] Hamad G.G., Choban P.S. (2005). Enoxaparin for thromboprophylaxis in morbidly obese patients undergoing bariatric surgery: findings of the prophylaxis Against VTE outcomes in bariatric surgery patients receiving enoxaparin (PROBE) study. Obes. Surg..

[33] Aminian A., Andalib A., Khorgami Z. (2017). Who should get extended thromboprophylaxis after bariatric surgery?: a risk assessment tool to guide indications for post-discharge pharmacoprophylaxis. Ann. Surg..

[34] Abuoglu H.H., Müftüoğlu M.A.T., Odabaşı M. (2019). A new protocol for venous thromboembolism prophylaxis in bariatric surgery,. Obes. Surg..

[35] Becattini C., Agnelli G., Manina G. (2012). Venous thromboembolism after laparoscopic bariatric surgery for morbid obesity: clinical burden and prevention. Surg. Obes. Relat. Dis..

[36] Angrisani L., Santonicola A., Iovino P. (2018). IFSO worldwide survey 2016: primary, endoluminal, and revisional procedures. Obes. Surg..

[37] Romano P.S., Mark D.H. (1994). Bias in the coding of hospital discharge data and its implications for quality assessment. Med. Care.

[38] Kniffin W.D., Baron J.A., Barrett J. (1994). The epidemiology of diagnosed pulmonary embolism and deep venous thrombosis in the elderly,. Arch. Intern. Med..

[39] White R.H., Zhou H., Romano P.S. (1998). Incidence of idiopathic deep venous thrombosis and secondary thromboembolism among ethnic groups in California. Ann. Intern. Med..

[40] Nguyen N.T., Hinojosa M.W., Fayad C. (2007). Laparoscopic surgery is associated with a lower incidence of venous thromboembolism compared with open surgery. Ann. Surg..

[41] Moussa O., Ardissino M., Tang A. (2019). Long-term impact of bariatric surgery on venous thromboembolic risk: a matched cohort study, [published online ahead of print, 2019 Dec 17]. Ann. Surg..

[42] Ammann E.M., Kalsekar I., Yoo A. (2019). Assessment of obesity prevalence and validity of obesity diagnoses coded in claims data for selected surgical populations: a retrospective, observational study,. Medicine (Baltim.).

[43] Clapp B., Liggett E., Phan C. (2020). Conversions to open surgery greatly increase complications: an analysis of the MBSAQIP database [published online ahead of print. 2020 Feb 10]. Surg Obes Relat Dis.

[44] Noyes K., Myneni A.A., Schwaitzberg S.D., Hoffman A.B. (2020). Quality of MBSAQIP data: bad luck, or lack of QA plan?. Surg. Endosc..

[45] Garrett M., Poppe K., Wooding A. (2020). Private and public bariatric surgery trends in New Zealand 2004-2017: Demographics, cardiovascular comorbidity and procedure selection, [published online ahead of print. 2020 Mar 12]. Obes Surg.

[46] Lim J., Cho Y.H., Yamamoto H. (2017). Governmental or social support of bariatric surgery in the Asia-Pacific region. J Obes Metab Syndr.

[47] Clough A., Hamill D., Jackson S. (2017). Outcome of three common bariatric procedures in the public sector. ANZ J. Surg..

[48] Kowalewski P.K., Rogula T.G., Lagardere A.O. (2019). Current practice of global bariatric tourism-survey-based study. Obes. Surg..

[49] Kruger R.S., Pricolo V.E., Streeter T.T. (2014). A bariatric surgery center of excellence: operative trends and long-term outcomes. J. Am. Coll. Surg..

[50] Sheppard C.E., Lester E.L., Chuck A.W. (2014). Medical tourism and bariatric surgery: who pays?. Surg. Endosc..

[51] McGrice M., Don Paul K. (2015). Interventions to improve long-term weight loss in patients following bariatric surgery: challenges and solutions. Diabetes Metab Syndr Obes.

[52] Kim D.H., Sheppard C.E., de Gara C.J. (2016). Financial costs and patients' perceptions of medical tourism in bariatric surgery. Can. J. Surg..

[53] Gagner M. (2017). Bariatric surgery tourism hidden costs? How Canada is not doing its part in covering bariatric surgery under the Canada Health Act. Can. J. Surg..

[54] Cowan G.S. (2000). Obligations of the bariatric surgeon. Obes. Surg..

[55] Cowan G.S. (1998). The Cancun IFSO Statement on bariatric surgeon qualifications. International federation for the surgery of obesity. Obes. Surg..

[56] Flum D.R., Salem L., Elrod J.A. (2005). Early mortality among Medicare beneficiaries undergoing bariatric surgical procedures. J. Am. Med. Assoc..

[57] Mechanick J.I., Youdim A., Jones D.B. (2013). Clinical practice guidelines for the perioperative nutritional, metabolic and nonsurgical support of the bariatric surgery patients 2013 update. Surg. Obes. Relat. Dis..

[58] Mechanick J.I., Kushner R.F., Sugerman H.J. (2009). American association of clinical endocrinologists; obesity society; American society for metabolic & bariatric surgery. American association of clinical endocrinologists, the obesity society, and American society for metabolic & bariatric surgery medical guidelines for clinical practice for the perioperative nutritional, metabolic, and nonsurgical support of the bariatric surgery patient, obesity. 17 Sup.

[59] Azagury D., Morton J.M. (2016). Bariatric surgery outcomes in US accredited vs non- accredited centers: a systematic review,. J. Am. Coll. Surg..

[60] Jafari M.D., Jafari F., Young M.T. (2013). Volume and outcome relationship in bariatric surgery in the laparoscopic era. Surg. Endosc..

[61] Readmissions Reduction Program http://www.cms.gov/Medi%20care/Medicare-Fee-for-%20Service-Payment/AcuteInpatientPPS/Readmissions-Reduction-Program.html.

[62] Rosenthal R.J. (2014). Readmissions after bariatric surgery: does operative technique and procedure choice matter?. Surg. Obes. Relat. Dis..

[63] Gargiulo I.N., Veith F.J., Lipsitz E.C. (2006). Experience with inferior vena cava filter placement in patients undergoing open gastric bypass procedures. J. Vasc. Surg..

[64] Melinek J., Livingston E., Cortina G. (2002). Autopsy findings following gastric bypass surgery for morbid obesity. Arch. Pathol. Lab Med..

[65] Kearon C., Julian J.A., Newman T.E. (1998). Noninvasive diagnosis of deep venous thrombosis. McMaster diagnostic imaging practice guidelines initiative. Ann. Intern. Med..

[66] Kearon C. (2001). Noninvasive diagnosis of deep vein thrombosis in postoperative patients. Semin. Thromb. Hemost..

[67] Gould J. (2017). Discussion of “Perioperative complications increase the risk of venous thromboembolism following bariatric surgery”. Am. J. Surg..

[68] Steele K.E., Schweitzer M.A., Prokopowicz G. (2011). The long-term risk of venous thromboembolism following bariatric surgery,. Obes. Surg..

[69] Jamal M.H., Corcelles R., Shimizu H. (2015). Thromboembolic events in bariatric surgery: a large multi-institutional referral center experience. Surg. Endosc..

[70] Helm M.C., Simon K., Higgins R. (2017). Perioperative complications increase the risk of venous thromboembolism following bariatric surgery. Am. J. Surg..

[71] Agency for Healthcare Research and Quality (AHRQ) Statistical brief #23. Bariatric Surgery Utilization and Outcomes in 1998 and 2004. http://www.hcup-us.ahrq.gov/reports/statbriefs/sb23.jsp.

[72] Smith M.D., Patterson E., Wahed A.S. (2011). Thirty-day mortality after bariatric surgery: independently adjudicated causes of death in the longitudinal assessment of bariatric surgery. Obes. Surg..

[73] Kerr T.M., Lutter K.S., Moeller D.M. (1990). Upper extremity venous thrombosis diagnosed by duplex scanning. Am. J. Surg..

[74] Prandoni P., Polistena P., Bernardi E. (1997). Upper-extremity deep vein thrombosis. Risk factors, diagnosis, and complications. Arch. Intern. Med..

[75] Johnson C.M., de la Torre R.A., Scott J.S. (2005). Mesenteric venous thrombosis after laparoscopic Roux-en-Y gastric bypass. Surg. Obes. Relat. Dis..

[76] De Roover A., Detry O., Coimbra C. (2006). Pylephlebitis of the portal vein complicating intra-gastric migration of an adjustable gastric band. Obes. Surg..

[77] Angrisani L., Santonicola A., Iovino P. (2017). Bariatric surgery and endoluminal procedures: IFSO worldwide survey 2014. Obes. Surg..

[78] Ozsoy Z., Demir E. (2018). Which bariatric procedure is the most popular in the world? A bibliometric comparison,. Obes. Surg..

[79] Shaheen O., Siejka J., Thatigotla B. (2017). A systematic review of portomesenteric vein thrombosis after sleeve gastrectomy,. Surg. Obes. Relat. Dis..

[80] Shoar S., Saber A.A., Rubenstein R. (2018). Portomesentric and splenic vein thrombosis (PMSVT) after bariatric surgery: a systematic review of 110 patients. Surg. Obes. Relat. Dis..

[81] El-Matbouly M.A., Khidir N., Sargsyan D. (2018). Portomesentric vein thrombosis: our experience of 3527 laparoscopic bariatric surgery procedures in Qatar. J. Obes. Bariatrics.

[82] Caruso F., Cesana G., Lomaglio L. (2017). Is portomesenteric vein thrombosis after laparoscopic sleeve gastrectomy related to short-course prophylaxis of thromboembolism? A monocentric retrospective analysis about an infrequent but not rare complication and review of the literature,. J. Laparoendosc. Adv. Surg. Tech..

[83] Shafik Y., Jaber T. (2018). Portal vein thrombosis occurring after laparoscopic sleeve gastrectomy: a short series and review of literature,. Saudi J Laparosc.

[84] Salinas J., Barros D., Salgado N. (2014). Portomesenteric vein thrombosis after laparoscopic sleeve gastrectomy. Surg. Endosc..

[85] Bani Hani M.N., Al Manasra A.R.A., Obeidat F. (2019). Portomesenteric venous thrombosis post-laparoscopic sleeve gastrectomy: do energy systems pose as instigating factor to this infrequent complication?. Clin. Med. Insights Case Rep..

[86] Szomstein S., Betancourt A. (2019). Mesenteric venous thrombosis following bariatric surgery—nursing accredited. Bariatric times. https://bariatrictimes.com/mesenteric-venous-thrombosis-bariatric-surgery/.

[87] Muneer M., Abdelrahman H., El-Menyar A. (2016). Portomesenteric vein thrombosis after laparoscopic sleeve gastrectomy: 3 case reports and a literature review. Am J Case Rep.

[88] Hutter M.H., Randall S., Khuri S.F. (2006). Lararoscopic versus open gastric bypass for morbid obesity. Ann. Surg..

[89] Mukherjee D., Lidor A.O., Chu K.M. (2008). Postoperative venous thromboembolism rates vary significantly after different types of major abdominal operations. J. Gastrointest. Surg..

[90] Prystowsky J.B., Morasch M.D., Eskandari M.K. (2005). Prospective analysis of the incidence of deep venous thrombosis in bariatric surgery patients. Surgery.

[91] Daigle C.R., Brethauer S.A., Tu C. (2018). Which postoperative complications matter most after bariatric surgery? Prioritizing quality improvement efforts to improve national outcomes. Surg. Obes. Relat. Dis..

[92] Sapala J.A., Wood M.H., Schuhknecht M.P. (2003). Fatal pulmonary embolism after bariatric operations for morbid obesity: a 24-year retrospective analysis. Obes. Surg..

[93] Morino M., Toppino M., Forestieri P. (2007). Mortality after bariatric surgery: analysis of 13,871 morbidly obese patients from a national registry. Ann. Surg..

[94] Livingston E.H., Huerta S., Arthur D. (2002). Male gender is a predictor of morbidity and age a predictor of mortality for patients undergoing gastric bypass surgery. Ann. Surg..

[95] Dang J.T., Switzer N., Delisle M. (2019). Predicting venous thromboembolism following laparoscopic bariatric surgery: Development of the BariClot tool using the MBSAQIP database. Surg. Endosc..

[96] Hamad G.G., Bergqvist D. (2007). Venous thromboembolism in bariatric surgery patients: an update of risk and prevention. Surg. Obes. Relat. Dis..

[97] Omalu B.I., Ives D.G., Buhari A.M. (2007). Death rates and causes of death after bariatric surgery for Pennsylvania residents, 1995 to 2004. Arch. Surg..

[98] Parker S.G., McGlone E.R., Knight W.R. (2015). Enoxaparin venous thromboembolism prophylaxis in bariatric surgery: a best evidence topic,. Int. J. Surg..

[99] Khan S., Rock K., Baskara A. (2016). Trends in bariatric surgery from 2008 to 2012. Am. J. Surg..

[100] Brasil Ministério da Saúde e Ministério do Planejamento [Internet] IBGE (2010). Pesquisa de orçamentos familiares (2008-2009) – Antropometria e estado nutri- cional de crianças, adolecentes e adultos no Brasil. http://www.ibge.gov.br/home/estatistica/populacao/condicaodevida/pof/2008_2009_encaa/pof_20082009_encaa.pdf.

[101] World Health Organization Obesity and overweight (2020). https://www.who.int/news-room/fact-sheets/detail/obesity-and-overweight.

[102] Canadian Institute for Health Information (2014). Bariatric surgery in Canada. Ottawa: CIHI.

[103] SOREG Scandinavian Obesity Surgery registry Sweden http://www.ucr.uu.se/soreg/.

[104] Burns E.M., Naseem H., Bottle A. (2010). Introduction of laparoscopic bariatric surgery in England: observational population cohort study. BMJ.

[105] Lazzati A., Guy-Lachuer R., Delaunay V. (2014). Bariatric surgery trends in France: 2005-2011. Surg. Obes. Relat. Dis..

[106] Kaplan U., Romano-Zelekha O., Goitein D. (2020). Trends in bariatric surgery: a 5-year analysis of the Israel national bariatric surgery registry. Obes. Surg..

[107] Buchwald H., Avidor Y., E Braunwald (2004). Bariatric surgery: a systematic review and meta-analysis. J. Am. Med. Assoc..

[108] Eren S.K., Çınar O., Yalı A. (2018). Portomesenteric and splenic vein thrombosis after laparoscopic sleeve gastrectomy. Laparosc Endosc Surg Sci.

[109] Weller W.E., Rosati C. (2008). Comparing outcomes of laparoscopic versus open bariatric surgery. Ann. Surg..

[110] Sekhar N., Torquati A., Youssef Y. (2007). A comparison of 399 open and 568 laparoscopic gastric bypasses performed during a 4-year period,. Surg. Endosc..

[111] Suter M., Giusti V., Heraief E. (1999). Early results of laparoscopic gastric banding compared with open vertical banded gastroplastym. Obes. Surg..

[112] Kearon C., Akl E.A., Comerota A.J. (2012). American college of chest physicians. Antithrombotic therapy for VTE disease: antithrombotic therapy and prevention of thrombosis, 9th ed: American college of chest physicians evidence-based clinical practice guidelines. Chest.

[113] Sugerman H.J., Sugerman E.L., Wolfe L. (2001). Risks and benefits of gastric bypass in morbidly obese patients with severe venous stasis disease. Ann. Surg..

[114] Gaszynski T., Szewczyk T. (2014). The influence of laparoscopic vs. open gastric bypass on hemodynamic function in morbidly obese patients during general anesthesia,. Wideochir Inne Tech Maloinwazyjne.

[115] Tian H.L., Tian J.H., Yang K.H. (2011). The effects of laparoscopic vs. open gastric bypass for morbid obesity: a systematic review and meta-analysis of randomized controlled trials,. Obes. Rev..

[116] Reoch J., Mottillo S., Shimony A. (2011). Safety of laparoscopic vs open bariatric surgery: a systematic review and meta-analysis. Arch. Surg..

[117] Winegar D.A., Sherif B., Pate V., DeMaria E.J. (2011). Venous thromboembolism after bariatric surgery performed by bariatric surgery center of excellence Participants: analysis of the bariatric outcomes longitudinal database. Surg. Obes. Relat. Dis..

[118] Aarts M., Okrainec A., Glicksman A. (2011). Adoption of enhanced recovery after surgery (ERAS) strategies for colorectal surgery at academic teaching hospitals and impact on total length of hospital stay. Surg. Endosc..

[119] Greenblatt D.Y., Rajamanickam V., Pugely A.J. (2012). Short-term outcomes after laparoscopic-assisted proctectomy for rectal cancer: results from the ACS NSQIP. J. Am. Coll. Surg..

[120] Schwartz M.L., Drew R.L., Chazin-Caldie M. (2004). Factors determining conversion from laparoscopic to open Roux-en-Y gastric bypass. Obes. Surg..

[121] Higa K.D., Boone K.B., Ho T. (2000). Laparoscopic Roux- en-Y gastric bypass in morbid obesity: technique and preliminary results of our first 400 patients. Arch. Surg..

[122] Schauer P.R., Ikramuddin S., Gourash W. (2000). Outcomes after laparoscopic Roux-en- Y gastric bypass for morbid obesity. Ann. Surg..

[123] Eagon J.C., Marin D. (2003). Laparoscopic approach decreases hernia stomal stenoses and overall complications in gastric bypass. Obes. Surg..

[124] Papasavas P.K., Caushaj P.F., McCormick J.T. (2003). Laparoscopic management of complications following laparoscopic Roux-en-Y gastric bypass for morbid obesity. Surg. Endosc..

[125] Rosenthal R., Mehran A., Brasesco O. (2003). Experience with 218 consecutive laparoscopic antecolic Roux-en-Y gastric bypass procedures. Obes. Surg..

[126] Nguyen N.T., Nguyen B., Shih A. (2013). Use of laparoscopy in general surgical operations at academic centers. Surg. Obes. Relat. Dis..

[127] Shimada Y.J., Gibo K., Tsugawa Y. (2018). Bariatric surgery is associated with lower risk of acute care use for cardiovascular disease in obese adults. Cardiovasc. Res..

[128] Richardson W.S., Hamad G.G., Stefanidis D. (2017). SAGES VTE prophylaxis for laparoscopic surgery guidelines: an update. Surg. Endosc..

[129] Bartlett M.A., Mauck K.F., Daniels P.R. (2015). Prevention of venous thromboembolism in patients undergoing bariatric surgery. Vasc. Health Risk Manag..

[130] Pivalizza E.G., Pivalizza P.J., Weavind L.M. (1997). Perioperative thromboelastography and sonoclot analysis in morbidly obese patients. Can. J. Anaesth..

[131] Caprini J.A. (2005). Thrombosis risk assessment as a guide to quality patient care. Dis Mon.

[132] Rocha A.T., de Vasconcellos A.G., da Luz Neto E.R. (2006). Risk of venous thromboembolism and efficacy of thromboprophylaxis in hospitalized obese medical patients and in obese patients undergoing bariatric surgery. Obes. Surg..

[133] Modasi A., Dang J.T., Afraz S. (2019). Bariatric surgery outcomes in patients on preoperative Therapeutic anticoagulation: an analysis of the 2015 to 2017 MBSAQIP. Obes. Surg..

[134] Stein P.D., Matta F., Embolism Pulmonary, Venous Deep (2013). Thrombosis following bariatric surgery. Obes. Surg..

[135] Holländer S.W., Sifft A., Hess S. (2015). Identifying the bariatric patient at risk for pulmonary embolism: prospective clinical trial using duplex sonography and blood screening. Obes. Surg..

[136] Douglas I.J., Bhaskaran K., Batterham R.L. (2015). Bariatric surgery in the United Kingdom: a cohort study of weight loss and clinical outcomes in routine clinical care. PLoS Med..

[137] Gero D., Muller X., Staiger R.D. (2020). How to establish benchmarks for surgical outcomes?: a checklist based on an international expert delphi consensus, [published online ahead of print. 2020 May 8]. Ann Surg.

[138] Hamadi R., Marlow C.F., Nassereddine S., Taher A., Finianos A. (2019). Bariatric venous thromboembolism prophylaxis: an update on the literature. Expet Rev. Hematol..

[139] Tsai A., Schumann R. (2016). Morbid obesity and perioperative complications. Curr. Opin. Anaesthesiol..

[140] Singh K., Podolsky E.R., Um S. (2012). Evaluating the safety and efficacy of BMI-based preoperative administration of low-molecular-weight heparin in morbidly obese patients undergoing Roux-en-Y gastric bypass surgery. Obes. Surg..

[141] Stein P.D., Goldman J. (2009). Obesity and thromboembolic disease. Clin. Chest Med..

[142] Stein P.D., Matta F., Goldman J. (2011). Obesity and pulmonary embolism: the mounting evidence of risk and the mortality paradox. Thromb. Res..

[143] Abraha I., Montedori A., Serraino D. (2018). Accuracy of administrative databases in detecting primary breast cancer diagnoses: a systematic review. BMJ Open.

[144] Abraha I., Orso M., Grilli P. (2014). The current state of validation of administrative healthcare databases in Italy: a systematic review,. Int. J. Stat. Med. Res..

[145] Lohr K.N., Institute of Medicine (US) Committee on Regional Health Data Networks; Donaldson MS (1994). Health databases and health database organizations: Uses, benefits, and concerns. Health Data in the Information Age: Use, Disclosure, and Privacy. Washington (DC): National Academies Press (US).

[146] Corbellini C., Andreoni B., Ansaloni L. (2018). Reliability and validity assessment of administrative databases in measuring the quality of rectal cancer management. Tumori.

[147] Scott J.D., Morton J.M., Brethauer S.A., DeMaria E.J., Kahan S., Hutter M.M., Accountable Care Organizations: A Primer (2019). Springer Nature, Cham Quality in Obesity Treatment.

[148] Snyder J.C., Silva D.S., Crooks V.A. (2016). Unpacking the financial costs of "bariatric tourism" gone wrong: who holds responsibility for costs to the Canadian health care system?. Can. J. Surg..

[149] Verde F., Alabi O., Prokopowicz G. (2018). Imaging modalities for detecting deep venous thrombosis after bariatric surgery. Curr Surg Rep.

[150] Khalifa I.G., Balamoun H.A., El Kaffas K. (2017). Value of pharmacologic thromboprophylaxis for prevention of thromboembolic complications in bariatric surgery. The Egyptian Journal of Surgery.

[151] Alenazi N.A., Ahmad K.S., Essa M.S. (2019). Porto-mesenteric vein thrombosis following laparoscopic sleeve gastrectomy for morbid obesity: case series and literature review. Int J Surg Case Rep.

